# Feasibility of uterine preservation in the management of early-stage uterine adenosarcomas: a single institute experience

**DOI:** 10.1186/s12957-017-1137-0

**Published:** 2017-04-19

**Authors:** Young-Jae Lee, Dae-Yeon Kim, Dae-Shik Suh, Jong-Hyeok Kim, Yong-Man Kim, Young-Tak Kim, Joo-Hyun Nam

**Affiliations:** Department of Obstetrics and Gynecology, University of Ulsan College of Medicine, Asan Medical Center, 88 Olympic-ro 43-gil, Songpa-gu, Seoul 138-736 South Korea

**Keywords:** Fertility preservation, Mullerian adenosarcoma of the uterus, Uterine adenosarcoma

## Abstract

**Background:**

We aimed to evaluate the efficacy and the safety of uterine preservation in patients with early-stage uterine adenosarcoma who want to preserve future fertility.

**Methods:**

We performed a retrospective review of patients with stage I uterine adenosarcoma diagnosed and treated at a single institute from 1998 through 2014.

**Results:**

Among the total of 31 patients, uterine preservation surgery was performed in 7 of the nulliparas. Of the 7 patients receiving uterine preservation surgery, 3 showed no evidence of disease (NED), 2 had persistent disease confined to the uterus, and 2 were alive with disease (AWD) after recurrence. One patient with an NED status had a vaginal delivery at term. In the uterine preservation group, 1 patient had sarcomatous overgrowth at the time of diagnosis and experienced disease recurrence. In the hysterectomy group, 3 of 24 patients had tumor recurrence. Of the five patients with tumor recurrence, four (80%) had sarcomatous overgrowth at diagnosis and it was significantly associated with recurrence by univariate analysis (OR 13.3, *p* = 0.027).

**Conclusions:**

Uterine preservation represents a possible treatment option for young female patients who want to maintain fertility. However, a detailed explanation of the risk of recurrence is necessary, especially in patients with sarcomatous overgrowth, which seems to be associated with a higher risk of recurrence.

**Trial registration:**

Retrospectively registered.

## Background

Mullerian adenosarcoma is a rare malignancy composed of benign epithelial and malignant stromal components and usually arises from the uterus [1]. Uterine adenosarcoma is considered to be a less aggressive disease than carcinosarcoma because its malignant component is usually low grade, and often a hysterectomy is curative [2]. The presence of sarcomatous overgrowth seems to be the factor most strongly associated with an aggressive clinical course, postoperative recurrence, metastasis, and a fatal outcome [3, 4]. The presence of heterologous elements, tumor grade, and myometrial invasion depth are other risk factors for metastasis [5].

Due to its rarity, there are limited data on the optimal therapy (i.e., primary surgery or adjuvant therapy) for uterine adenosarcoma. Total hysterectomy is generally considered the primary intervention for this disease, but uterine preservation is often desired in reproductive-age women. We aimed to analyze clinical outcomes according to which therapeutic methods were used in patients with early-stage uterine adenosarcoma to evaluate the efficacy of uterine preservation in patients wanting to preserve their future fertility.

## Methods

We performed a retrospective review of patients with stage I uterine adenosarcoma diagnosed and treated at Asan Medical Center, Seoul, Korea, from 1998 to 2014. Medical records were reviewed for age at diagnosis, largest tumor diameter, parity at diagnosis and last follow-up date, primary treatment, adjuvant therapy, recurrence, sarcomatous overgrowth, and disease status at last follow-up. All cases were re-staged according to International Federation of Gynecology and Obstetrics (FIGO) 2009 criteria (Table 1) [6], and only patients with stage I and uterine origin adenosarcoma were included. Some patients were assigned a clinical stage based on imaging reports and pathology because they did not undergo a complete staging procedure. Sarcomatous overgrowth was defined as the presence of pure sarcoma occupying at least 25% of the tumor without a benign glandular component. We obtained Institutional Review Board approval for these studies. The relationships between variable characteristics and recurrence were assessed by univariate analysis using Chi-square and Fisher’s exact tests and by multivariate analysis using logistic regression analysis to identify independent risk factors for recurrence. Recurrence distributions were assessed by the method of Kaplan and Meier. Descriptive statistics and data analysis were performed using SPSS ver.20.0 (SPSS Inc, Chicago, IL, USA).

**Table 1:** Characteristics of the 31 study patients with early-stage uterine adenosarcoma

Variables	No. of patients (%)
Age at diagnosis, median (range)	44.5 (21–81 years)
Largest tumor diameter, mean (range)	4.66 (0.8–22.0 cm)
FIGO 2009 stage	
IA	21 (67.7)
IB	8 (25.8)
IC	2 (6.5)
Sarcomatous overgrowth	
Present	10 (32.3)
Absent	21 (67.7)
Primary therapy	
No hysterectomy	7 (22.6)
Hysterectomy	24 (77.4)
Adjuvant therapy	
Chemotherapy	5 (16.1)
Radiotherapy	1 (3.2)
Hormone therapy	1 (3.2)
Total	31 (100%)

## Results

From 1998 to 2014, 33 patients were diagnosed with Mullerian adenosarcoma at our institution, with 31 meeting the inclusion criteria for our current study. One of the excluded patients had stage IV disease, and the other had ovarian adenosarcoma. The characteristics of the 31 study patients with early-stage uterine adenosarcoma are summarized in Table 2. The median age at diagnosis was 44.5 years, and the mean of the largest tumor diameter was 4.66 cm. Ten patients had adenosarcoma with sarcomatous overgrowth. Thirteen patients were under 40 years old at the time of diagnosis, and 9 of these were nulliparas. Uterine preservation surgery, such as hysteroscopic mass excision, cervical mass excision, or dilatation and curettage, was performed in 7 of the nulliparas. Laparoscopy-assisted vaginal hysterectomy or total abdominal hysterectomy was performed in 13 and 11 of the patients, respectively. Thirteen patients had a bilateral salpingo-oophorectomy (BSO) at the time of hysterectomy. Eleven patients underwent pelvic lymphadenectomy, while 3 patients underwent para-aortic lymphadenectomy. Lymph node metastasis was not identified in any patients. Five patients received adjuvant chemotherapy, 3 of them receiving ifosphamide with cisplatin and 2 receiving doxorubicin with cisplatin. Adjuvant radiation was performed in 1 patient, while hormone therapy was performed in 1 other patient.

**Table 2:** Clinical outcomes of the study patients with early-stage uterine adenosarcoma who received uterine preservation therapy

Number	Age at diagnosis	Stage	Marital status^a^	Primary therapy	SO	Adjuvant therapy	Recurrence	Time to recurrence, months	Disease status	F/U months	Event
1	33	IB	Single	HSC-mass excision	N	No	No		NED	12	
2	33	IA	Single	HSC-mass excision	N	MPA 3 months	No		NED	77	Vaginal delivery
3	40	IA	Single	D/C/Bx	N	No	No		NED	32	
4	21	IB	Single	HSC-mass excision	N	No	No		Persistent	17	
5	22	IA	Single	Cx mass excision	N	No	No		Persistent	38	
6	27	IA	Single	HSC-mass excision	Y	I/P#4	Yes	13	AWD	22	TAH c BSO d/t seeding
7	27	IB	Single	HSC-mass excision	N	No	Yes	27	AWD	38	

^a^Status at diagnosis

*SO* sarcomatous overgrowth, *F*/*U* follow-up, *HSC* hysteroscopic, *MPA* medroxy progesterone acetate, *D*/*C*/*Bx* dilatation and curettage biopsy, *Cx* cervix, *I*/*P* ifosphamide/cisplatin, *TAH c BSO* total abdominal hysterectomy with bilateral salpingo-oophorectomy, *d*/*t* due to, *NED* no evidence of disease, *AWD* alive with disease

Of the 7 patients in the uterine preservation group, 3 showed no evidence of disease (NED), 2 had persistent disease confined to uterus, and 2 were alive with disease (AWD) after recurrence. The median follow-up was 32 months (Table 3). In the uterine preservation group, 3 patients were virgins, 3 were single, and 1 was a married woman. After 17 months after primary therapy, the married woman had a vaginal delivery of a 2950-g female at 39 + 2 weeks of gestation. Two patients had persistent disease upon receiving routine oncological follow-up with ultrasonography or computed tomography and again received dilatation and curettage. In this group, the tumor lesion was confined to the uterus during the follow-up period. Two patients experienced disease recurrence. One patient received only observation of a suspicious small mass in the endometrial cavity because she decided not to continue conservative therapy or have a hysterectomy. Another patient underwent disease recurrence with peritoneal seeding 10 months after adjuvant chemotherapy (ifosphamide and cisplatin). She was the only nullipara with sarcomatous overgrowth at the time of diagnosis. She underwent total abdominal hysterectomy with BSO, pelvic lymph node dissection, lower anterior resection, and mass excision.

**Table 3:** FIGO criteria (2009) for Mullerian adenosarcomas

Stage	Definition
I	Tumor limited to uterus
IA	*Tumor limited to endometrium and endocervix* with no myometrial invasion
IB	Less than or equal to half myometrial invasion
IC	More than half myometrial invasion
II	Tumor extends beyond the uterus, within the pelvis
IIA	Adnexal involvement
IIB	Tumor extends to extrauterine pelvic tissue
III	Tumor invades abdominal tissues (not just protruding into the abdomen)
IIIA	One site
IIIB	More than one site
IIIC	Metastasis to pelvic and or para-aortic lymph nodes
IV	
IVA	Tumor invades bladder and or rectum
IVB	Distant metastasis

**Fig. 1:**
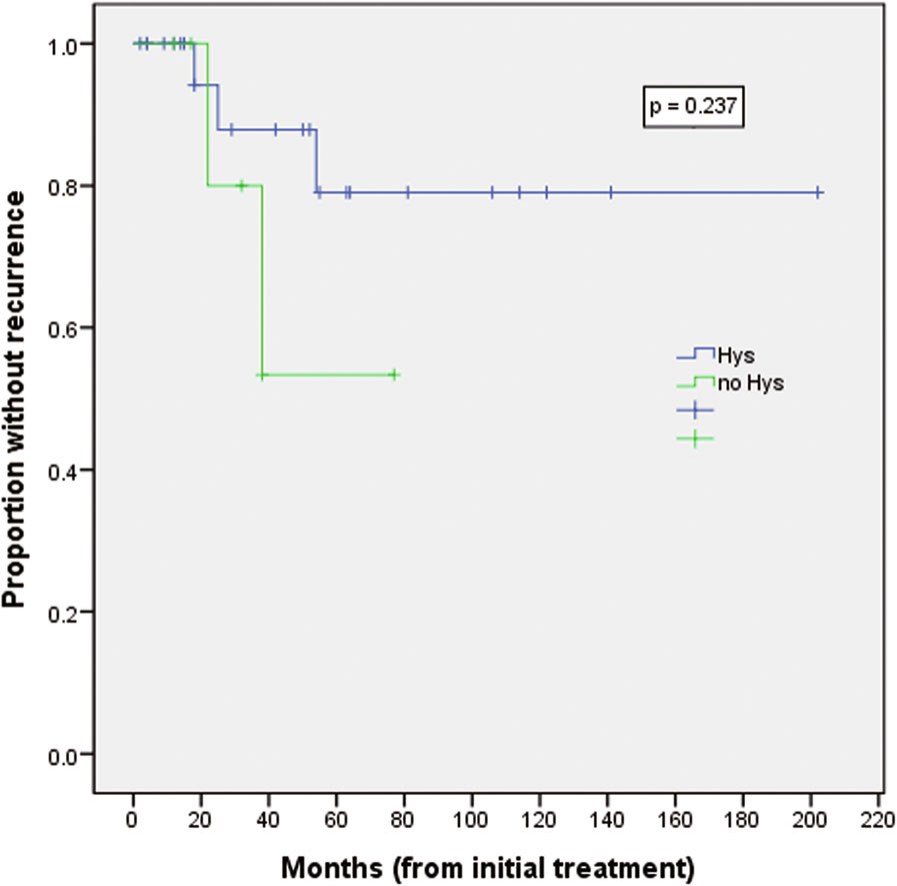
Kaplan-Meier curves for recurrence-free survival in patients with uterine adenosarcoma with and without hysterectomy

In the hysterectomy group, 3 of 24 patients had tumor recurrence. One of these patients was a nullipara with sarcomatous overgrowth who received a laparoscopic hysterectomy with bilateral salpingectomy and pelvic lymphadenectomy at another hospital. However, she had disease recurrence after 5 months and returned to our hospital. She received pelvic mass excision with total omentectomy and chemotherapy for 6 cycles (ifosphamide and cisplatin). Another 2 patients in the hysterectomy group with tumor recurrence had sarcomatous overgrowth at diagnosis and recurred at 18 and 48 months after hysterectomy, respectively. They could not receive chemotherapy due to old age and general prostration. In our total adenosarcoma patient series of 31 cases, 5 (16.1%) had tumor recurrence. Four of these 5 (80%) had sarcomatous overgrowth at diagnosis. In the non-sarcomatous overgrowth group, 1 of 21 patients (4.8%) had tumor recurrence. In the sarcomatous overgrowth group, 4 of 10 patients (40%) had tumor recurrence. Sarcomatous overgrowth was significantly associated with recurrence by univariate analysis (OR 13.3, 95% CI 1.24–143.15, *p* = 0.027) (Table 4). Hysterectomy (OR 2.8, 95% CI 0.37–21.49, *p* = 0.562) and adjuvant chemotherapy (OR 0.52, 95% CI 0.04–6.36, *p* = 0.525) were not found to be significantly associated with recurrence. By multivariate analysis, there were no factors found to be associated with recurrence. Recurrence-free survival analysis using the method of Kaplan and Meier revealed no statistically significant differences between patients with or without hysterectomy (*p* = 0.237; Fig. 1).

**Table 4:** Univariate and multivariate analysis for factors associated with uterine adenosarcoma recurrence

	Univariate analysis		Multivariate analysis	
Variables	Odds ratio (95% CI)	*p* value	Odds ratio (95% CI)	*p* value
Sarcomatous overgrowth	13.3 (1.24–143.15)	0.027	4.436 (0.414–47.481)	0.218
Hysterectomy	2.8 (0.37–21.49)	0.562	2.247 (0.171–29.588)	0.538
Bilateral salpingo-oophorectomy	1.10 (0.156–7.740)	0.659		
Adjuvant chemotherapy	0.52 (0.04–6.36)	0.525		

*CI* confidence interval

## Discussion

Removal or destruction of sexual organs is used as a primary therapy in most cases of gynecological malignancies originating from reproductive organs, such as the uterus, ovaries, fallopian tube, vulva, and vagina. However, destructive surgery or radiation can result in abrupt onset of menopause, fertility loss, sexual function decline, pelvic pain, depression, and anxiety. Because of these factors, careful counseling is needed when making a decision about these therapeutic methods. For uterine adenosarcoma, total hysterectomy with bilateral salpingo-oophorectomy is considered the standard primary therapy. However, in young patients with uterine adenosarcoma, surgery that spares fertility should be considered, as the malignant component is usually low grade, leading to a good prognosis. In the largest published study to date, only 2 out of 100 patients had adnexal involvement, and both of these had grossly abnormal adnexa [7]. Given the low reported rates of ovarian involvement, ovarian preservation in patients of reproductive age with uterine adenosarcoma seems to be a reasonable option in the absence of gross metastasis [2, 8]. However, there are limited data on the safety and effectiveness of uterine-preserving surgery in patients with uterine adenosarcoma.

In our report, 7 of 9 nullipara received uterine preservation therapy, such as hysteroscopic mass excision, cervical mass excision, or dilatation and curettage. Of these 7 patients, 3 showed NED, 2 had persistent disease confined to the uterus, and 2 were AWD after recurrence at a median follow-up of 32 months. One patient with NED status was married after her diagnosis and went on to have a vaginal delivery 17 months after primary therapy. Therefore, 1 of 1 patient (100%) in the uterine preservation group who tried to get pregnant was successful. Two patients with persistent disease and 1 patient with disease recurrence had tumor lesions confined to the uterus during the follow-up period. Therefore, at a median follow-up of 32 months, 6 of 7 patients (85.7%) in the uterine preservation group still have the possibility of a future pregnancy.

The relationship of sarcomatous overgrowth in patients with uterine adenosarcoma to poor prognosis was identified by past articles [2, 4, 5, 9], (Table 5). In the recurrence group, the percentage of sarcomatous overgrowth presentation was increased in all articles. In our present study, one patient in the uterine preservation group had sarcomatous overgrowth at diagnosis. In spite of adjuvant chemotherapy with 4 cycles of ifosphamide and cisplatin, she had disease recurrence with peritoneal seeding 13 months after primary therapy. In our hysterectomy group, 9 patients had sarcomatous overgrowth, and 3 patients recurred. This recurrence risk (40%) is far greater than the risk in the non-sarcomatous overgrowth group (4.8%). By univariate analysis, sarcomatous overgrowth was significantly associated with recurrence (OR 13.3, 95% CI 1.24–143.15, *p* = 0.027). However, this relationship was not demonstrated by multivariate analysis (*p* = 0.218). This discrepancy is likely due to the small sample size of the study and might be resolved by a future study with a larger sample size. The role of the adjuvant therapies like chemotherapy, radiotherapy, and hormonal therapy after primary surgery of uterine adenosarcoma is still not established because of data limitations. In a recent study, no specific adjuvant therapy led to a significant improvement in progression-free survival or overall survival [2]. In our present study, adjuvant chemotherapy was not associated with recurrence rates.

**Table 5:** Characteristics and clinical outcomes of uterine adenosarcoma (literature review and present study)

Source	No. of patients	Age	Stage I of FIGO classification	SO	Recurrence	SO + recurrence	Time to recurrence^a^	F/U months
Caroll, et al. [2]	74	54	59 (80%)	34 (42%)	34 (46%)	24 (71%)	18.3	56.5
Tanner et al. [5]	19	55	15 (79%)	5 (26%)	5 (26%)	4 (80%)	20.1	72.9
Bernard et al. [4]	64	61	47 (73%)	30 (47%)	16 (36%)	11 (69%)	21.2	23.8
Kaku et al. [9]	31	ND	ND	17 (55%)	9 (30%)	ND	ND	38.3
Present study	31	44.5	31 (100%)	10 (32%)	5 (16%)	4 (80%)	21.6	32.0

^a^Months

*SO* sarcomatous overgrowth, *F*/*U* follow-up, *ND* not defined

## Conclusions

To our knowledge, our current report is the first study to focus on uterine preservation in young patients with uterine adenosarcoma. Uterine preservation represents a possible treatment option for carefully screened young female patients who want to maintain fertility. However, a detailed explanation of the risks and benefits of the treatment alternatives and a strict oncological follow-up are necessary. Uterine preservation therapy might be risky, especially in patients with sarcomatous overgrowth, and definitive treatment such as hysterectomy seems to be a better choice. More data concerning the long-term outcomes of uterine preservation and adjuvant therapy are required in the future.
